# The use of targeted genomic capture and massively parallel sequencing in diagnosis of Chinese Leukoencephalopathies

**DOI:** 10.1038/srep35936

**Published:** 2016-10-25

**Authors:** Xiaole Wang, Fang He, Fei Yin, Chao Chen, Liwen Wu, Lifen Yang, Jing Peng

**Affiliations:** 1 Department of Pediatrics, Xiangya Hospital, Central South University, 87 Xiangya Road, Changsha, Hunan Province, China; 2 Hunan Intellectual and Developmental Disabilities Research Center, Pediatrics, 87 Xiangya Road, Changsha, Hunan Province, China; 3 State Key Laboratory of Medical Genetics, Central South University, 110 Xiangya Road, Changsha, Hunan Province, China.

## Abstract

Leukoencephalopathies are diseases with high clinical heterogeneity. In clinical work, it’s difficult for doctors to make a definite etiological diagnosis. Here, we designed a custom probe library which contains the known pathogenic genes reported to be associated with Leukoencephalopathies, and performed targeted gene capture and massively parallel sequencing (MPS) among 49 Chinese patients who has white matter damage as the main imaging changes, and made the validation by Sanger sequencing for the probands’ parents. As result, a total of 40.8% (20/49) of the patients identified pathogenic mutations, including four associated with metachromatic leukodystrophy, three associated with vanishing white matter leukoencephalopathy, three associated with mitochondrial complex I deficiency, one associated with Globoid cell leukodystrophy (or Krabbe diseases), three associated with megalencephalic leukoencephalopathy with subcortical cysts, two associated with Pelizaeus-Merzbacher disease, two associated with X-linked adrenoleukodystrophy, one associated with Zellweger syndrome and one associated with Alexander disease. Targeted capture and MPS enables to identify mutations of all classes causing leukoencephalopathy. Our study combines targeted capture and MPS technology with clinical and genetic diagnosis and highlights its usefulness for rapid and comprehensive genetic testing in the clinical setting. This method will also expand our knowledge of the genetic and clinical spectra of leukoencephalopathy.

Leukoencephalopathies are disorders that primarily affect the white matter of the central nervous system (CNS). It contains acquired leukoencephalopathy^1^,^2^,^3^ (leukoencephalopathy induced by ischemia, hypoxia, intoxication, infection, traumatic brain injuries, etc.), genetic leukoencephalopathy^4^,^5^,^6^ (such as metachromatic leukodystrophy, globoid cell leukodystrophy, X-linked adrenoleukodystrophy, etc.) In addition, it also contains some mitochondrial diseases, cerebral cortical degenerative disorders, and so on. Clinically, after considering clinical history, symptoms and brain MRI features, doctors may be able to give a diagnosis for acquired leukoencephalopathies. However, leukoencephalopathy is a disease with high clinical heterogeneity and may involve in multiple genes, it is difficult even for experienced neurologists to make definite diagnosis^7^,^8^,^9^. Therefore, we are in urgent need of finding an efficient, economical, and practical method for diagnosing leukoencephalopathies.

In recent years, gene sequencing technology got amazing advancement. Whole exome sequencing (WES) represents a significant breakthrough in clinical genetic as a powerful tool for etiological discovery in many kinds of disorders^10^. Benefited from the WES technology, a lot more pathogenic genes have been found and many types of diseases have been identified^11^,^12^,^13^. Innovative application of new technologies is one of the major factors driving advances in medical science, most clinical applications of next-generation sequencing (NGS) concentrate on known and potential candidate genes to generate clear reports and finally promote clinical diagnosis^14^,^15^,^16^,^17^,^18^. Targeted gene capture and massively parallel sequencing (MPS) have been shown to be an effective technique for genetic analysis and have already led to many exciting discoveries^19^,^20^. To make a clear or definite diagnosis for those patients with leukoencephalopathies, we designed a custom probe library containing 118 genes reported to be associated with leukoencephalopathies (Table 1).

We embarked on this study to assess the utility and effectiveness of targeted capture and MPS technology in 49 Chinese leukoencephalopathy patients. To our knowledge, this is the first study to use targeted gene capture and sequencing for leukoencephalopathies. 40.8% positive rate confirmed that the implementation of this method can accelerate diagnosis, reduce overall cost, and expand our knowledge of the genetic and clinical spectra of leukoencephalopathies.

## Results

### Demographic and Clinical characteristics of the total 49 patients

We summarized the clinical characteristics of the total 49 patients enrolled in this study and found 39 are male and 10 are female. The age at onset of symptoms varied from 20 days to 7 years and the average onset age was almost 1.2 years. The main neurologic complaint of these patients include developmental delay/regression (27/49, 55.1%), epilepsy (15/49, 30.6%), weakness (7/49, 14.3%), ataxia (5/49, 10.3%) and dystonia (5/49, 10.3%). The severity of the disease course is reflected in the developmental milestones achieved. Two patients have suspected familial clustering. One has been diagnosed as adrenoleukodystrophy by gene testing. His mother’s elder brother had the same clinic feature and MRI findings, and died at his age of 12. The other one has been diagnosed as mitochondrial complex I deficiency, and his elder sister has a similar brain MRI changes without significant neurological disease manifestation. The wide spectrum of MRI findings was noted in the study. Abnormality in periventricular, subcortical white matter and cerebellar hemisphere were common. Lumbar puncture and CSF analysis were performed in 19 patients. None of them had a positive result.

Twenty patients were identified pathogenic mutations in this study, and their demographic and clinical characteristics were shown in Table 2. However, more than half (29/49, 59%) of patients in our study did not reach the diagnosis.

### Targeted capture and MPS sequencing results

**Figure 1:**
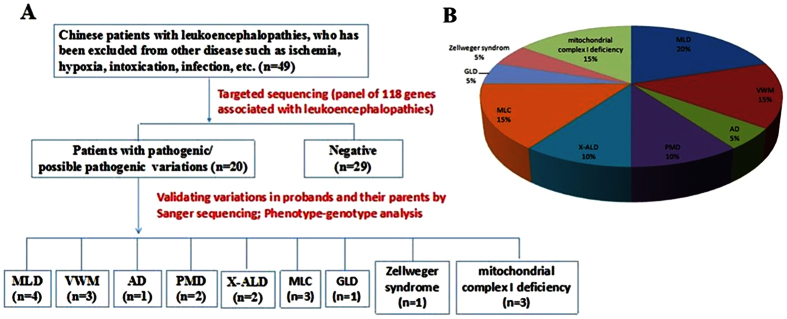
The etiology composition of Leukoencephalopathies in this cohort. (**A**) Flow diagram to exhibit workflow and results in this cohort. (**B**) Pie chart to exhibit the etiology composition of leukoencephalopathies in this cohort. MLD: metachromatic leukodystrophy, VWM: vanishing white matter disorder, AD: Alexander disease, PMD: Pelizaeus-Merzbacher Disease, X-ALD: X-linked adrenoleukodystrophy, MLC: megalencephalic leukoencephalopathy with subcortical cysts, GLD: globoid cell leukodystrophy.

In this study, 40.8% (20/49) exhibited pathogenic mutations, in which fifteen pathogenic variation sites have not yet been reported in HGMD. The proportion of each kind of disease diagnosed in our study is shown in Fig. 1. The most common disease diagnoses were metachromatic leukodystrophy (4/49, 8.2%), mitochondrial diseases (3/49, 6.1%), vanishing white matter disorder (3/49, 6.1%) and megalencephalic leukoencephalopathy with subcortical cysts (3/49, 6.1%). Details genetic data were summarized in Table 3.

## Discussion

With the widespread use of imaging examinations in nervous system diseases, finding the pathogeny of cerebral white matter lesions becomes an important clinical clue for neurologists. Because of the strong heterogeneity of hereditary leukoencephalopathy, it is difficult even for experienced doctors to make a definitive diagnosis, and a multistep process is often needed^7^,^21^. Currently, routine clinical diagnostic tests for leukodystrophy often consist of screening for genes on the basis of ethnic origin, MRI features, family history, personal history and findings from physical examinations^22^. In China, the problem seems more serious, with the lack of a referral system, many patients and their families wasted valuable time, finances, and medical resources seeing various doctors and getting repeat examinations in search of a correct diagnosis. Some patients who could even be cured missed the opportunity for effective treatment. However, due to the high cost of Sanger sequencing for the long list of candidate genes, more effective genetic screening methods are needed.

In recent years, targeted capture and MPS technologies have been widely used in clinical practice and have got satisfactory results^15^,^23^,^24^,^25^,^26^,^27^,^28^. To this end, we designed the gene panel contains 118 genes which are reported to be associated with leukoencephalopathies, not only contains genes associated with genetic leukoencephalopathy, but also mitochondrial disease, cerebral cortical degenerative disorders, etc. associated genes. Then we designed the probe library and performed this study to assess the utility and effectiveness of targeted capture and MPS in diagnosing leukoencephalopathy patients.

In our study, 40.8% (20/49) of the patients detected pathogenic mutations, which is higher than that of other commercially available chips. In our department, the positive rate of a mitochondrial disease chip is only 9.5%, and that of a metabolic disease chip is 16% (data not shown). These differences may be explained by the variety of pathogenic mutations and lack of a specific clinical phenotype associated with these disorders. Moreover, the results achieved using the leukoencephalopathy probe library may be explained by the distinctive brain MRI patterns that characterize leukoencephalopathy that was seen in most of the patients, providing a guide in the diagnostic process. In addition, patients had been thoroughly examined before the screening for leukoencephalopathy-associated genes, and other secondary causes were already excluded.

**Figure 2:**
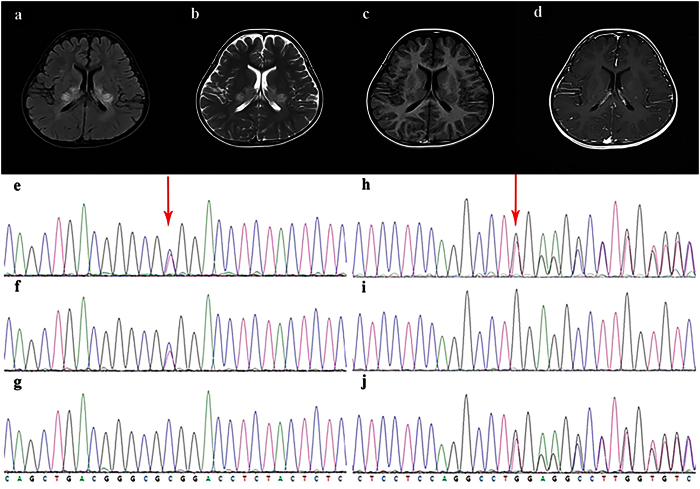
Brain MRI changes of case19 and the electropherogram of Sanger sequencing of the compound mutation of PEX6 gene. On FLAIR and T2-weighted sequences, abnormal hyperintense is seen in the splenium of corpus callosum, adjacent parieto-occipital white matter, posterior limbs of internal capsules extending to centrum oval, thalami and upper cervical spinal cord. Gadolinium enhancement is visible on T1-weighted sequences in internal capsules and anterior commissure. The child detected missense mutation on chr6:42932599(c.2735C > T) and nonsense mutation on chr6:42937459(c.1313insT), which were respectively inherited from the parents. (**a**) Brain MRI T1-weighted image. (**b**) Brain MRI T2-weighted image. (**c**) Brain MRI flare image. (**d**) Brain MRI enhanced image. (**e–g**) The electropherogram of Sanger sequencing of the probands (**e**), the father (**f**) and the mother (**g**) on chr6:42932599. (**h–j**) The electropherogram of Sanger sequencing of the probands (**h**), the father (**i**) and the mother (**j**) on chr6:42937459.

Among the result, one patient (case 19) was diagnosed with Zellweger syndrome with *PEX6* gene compound heterozygous mutations, *PEX6* gene mutation is reported to be associated with Peroxisome biogenesis disorder 4A/B^29^,^30^. The patient in our study was a 5.9-year-old girl exhibiting mental and motor retardation for 5 years, and with deterioration for 3 months (Clinical features and auxiliary examinations are included in Table 2). Brain MRI showed symmetrically increased signal intensity in T2-weighted images with gadolinium enhancement in the posterior limbs of the internal capsules (Fig. 2). There was no diffuse restriction or gadolinium enhancement in the periventricular area and deep white matter, similar to the features of X- ALD^31^,^32^. However, this is a female and the *ABCD1* gene in this patient exhibited a normal sequence and gene dosage. Given the diagnostic uncertainty, targeted capture and MPS were performed. Molecular testing identified *PEX6* gene compound heterozygous mutations, supporting the Zellweger spectrum disorder diagnosis in this patient. Genetic analysis showed that the two mutation sites were respectively inherited from the parents. The result showed us the effectiveness of this targeted capture and MPS method for the diagnosis of leukoencephalopathies.

The targeted capture and MPS method can not only diagnose genetic leukoencephalopathies, but also can make the diagnosis of mitochondrial diseases with white matter abnormal as the primary imaging changes. In our study, three of our patients had pathogenic gene mutations associated with mitochondrial complex I deficiency. Our team was the first to report leukoencephalopathy associated with mitochondrial complex I deficiency due to a novel mutation in the *NDUFAF1* gene (c.278A > G; c.247G > A)^33^. Mitochondrial complex I deficiency is the most frequent cause of respiratory chain defects in childhood, which accounts for various clinical presentations^34^,^35^. As the report, mutations have been described in 28 of these, including the 7 mitochondrial genes and 21 nuclear genes. Brain lesions caused by mitochondrial complex 1 deficiency are usually located in the brainstem, periaqueductal gray matter, the thalamus, etc. While, diffuse supratentorial leukoencephalopathy involving the deep lobar white matter may also occur in patients with mitochondrial complex 1 deficiency, especially in patients with nuclear DNA (nDNA) mutations. Some patients were available with abnormal white matter containing cysts in FLAIR sequences, and other patients may have notably hyperintense on T2 and very hypointense on T1 weighted images, suggesting cysts^33^,^36^. Therefore, containing pathogenic genes associated with mitochondrial diseases can promote the diagnosis of patients with leukoencephalopathies.

The patients in our study came from six provinces in central-south China. Therefore, the results may represent the specific disease incidence in this region. When clinicians encounter children with prominent cerebral white matter lesions that can’t be explained by a certain disease, application of leukoencephalopathy probe library gene screening may be useful. Targeted capture and MPS can detect multiple candidate genes at the same time in a fast, cost-effective way, and can facilitate clinical diagnosis. Moreover, by reaching a definitive diagnosis for children with leukoencephalopathy, we can better judge the prognosis for patients and provide genetic counseling.

In summary, our data demonstrate that the use of targeted capture and MPS technology coupled with NGS has great promise as a tool for screening leukoencephalopathy-related genes for diagnostic purposes in patients. At the same time, genetic testing results combined with detailed clinical phenotypes help us expand our knowledge of the clinical spectra of each type of leukoencephalopathy. This method enables clinicians to identify leukoencephalopathy even the clinical performance is not typical. Moreover, the entire process of targeted capture, sequencing, analysis, and parental analysis was rapid (requiring only 10 days for up to 12 patients).

While targeted genomic capture and MPS technology also has its limitation, it can only identify the known pathogenic mutations. With the development of gene testing technology, a lot more pathogenic mutations will be detected, so the panel should be renewed with the latest findings, and the patients with negative results of genetic testing can be re-tested using the newest panel. With the fast development of NGS sequencing, the price will be more accessible, we can choose whole exome sequencing (WES) if the targeted analysis is unrevealing, or we can directly choose the WES technology. WES will be the inevitable trend, but under the condition of most countries and before this come true, our panel with cheap price, fast testing speed and strong pertinence, still have the irreplaceable advantage. Thus, we expect that this method can serve as an inspirational starting point. This technology will enable us to conduct straightforward, comprehensive screening for more known leukoencephalopathy-related genes, and to expand and redefine the genetic and clinical spectra of leukoencephalopathies.

## Methods

### Patients

From December 2013 to December 2015, 49 patients (10 female and 39 male) were recruited into our cohort. All of these patients have white matter damage as the most obvious imaging characteristic. Two pediatric neurologists and one radiologist made the decisions together, according to the medical history, family history, physical examination and magnetic resonance imaging (MRI), patients with obvious ischemia, hypoxia, intoxication or infection was not enrolled in our cohort. The study design was approved by institutional review board of Xiangya Hospital of Central South University, China. And the study procedures were carried out in accordance with the requirements of regulations and procedures regarding human subject protection laws. After obtaining informed consent from all participants, we recorded the clinical features of the patients and collected blood samples from the patients and their parents via venipuncture.

### Panel design

We searched the OMIM and HGMD professional databases for genes which are reported to be associated with leukoencephalopathies. A custom-based targeted Agilent SureSelect pull-down panel was designed with the SureDesign program (Agilent Technologies). This target was 0.7 Mb of sequence from the coding exons (GRCh37/hg19 human reference sequence, UCSC Genome Browser) of 118 related candidate or known genes.

### Genetic testing

Genomic DNA was isolated from peripheral blood leukocytes (Promega, Beijing). Target-fragments are capture by SureDesign target enrichment kit (Agilent, Santa Clara, CA) and high throughput sequencing by HiSeq2500 sequencer (Illumina Inc, San Diego, CA) were conducted in house. Overall, 49 samples were sequenced pre lane and the mean depth is 583X.

### Bioinformatic Pipeline

For the quality control, the Cutadapt and FastQC were used to remove 3′-/5′- adapters andlow-quality reads, respectively. The clean reads were mapped to the reference human genome using the BWA (Burrows–Wheeler Aligner) program with at most two mismatches. The alignment files (bam) were generated with SAM tools and the reads of low mapping quality (<Q30) were filtered out. Clonal duplicated reads that may be derived from PCR artifacts were removed using Picard Tools by default parameters. Short read alignment and annotation visualization were performed using the IGV (Integrative Genomics Viewer). The percentage of alignment of the clean read to the exome regions was obtained using our custom Perl scripts on the base of alignment files. SNVs and indels were detected by GATK (Genome Analysis ToolKit). Comprehensive annotation of all of the detected SNVs and indels were annotated by ANNOVAR, including function implication (gene region, functional effect, mRNA GenBank accession number, amino acid change, cytoband, etc.) and allele frequency in 1000 Genomes, ExAc. Damaging missense mutations were predicted by SIFT, PolyPhen-2 and MutationTaster.

## Additional Information

**How to cite this article**: Wang, X. *et al*. The use of targeted genomic capture and massively parallel sequencing in diagnose of Chinese Leukoencephalopathies. *Sci. Rep.* **6**, 35936; doi: 10.1038/srep35936 (2016).

## Figures and Tables

**Table 1:** 118 genes targeted fo capture and sequencing.

Gene	NM number	Chromosome	Exons	Gene	NM number	Chromosome	Exons
ABAT	NM_001127448	chr16	16	FTL	NM_000146	chr19	4
ABCD1	NM_000033	chrX	10	FUCA1	NM_000147	chr1	8
ACOX1	NM_004035	chr17	14	GALC	NM_000153	chr14	17
ADGRG1	NM_001290143	chr16	14	GAN	NM_022041	chr16	11
AIMP1	NM_001142416	chr4	7	GCDH	NM_000159	chr19	12
ALDH3A2	NM_001031806	chr17	11	GFAP	NM_001242376	chr17	7
AMACR	NM_014324	chr5	5	GJA1	NM_000165	chr6	2
APP	NM_001136129	chr21	15	GJB1	NM_001097642	chrX	2
ARSA	NM_001085428	chr22	8	GJC2	NM_020435	chr1	2
ARSE	NM_000047	chrX	11	HEPACAM	NM_152722	chr11	7
ASPA	NM_000049	chr17	6	HSPD1	NM_199440	chr2	12
ATP13A2	NM_001141974	chr1	27	HTRA1	NM_002775	chr10	9
AUH	NM_001698	chr9	10	L2HGDH	NM_024884	chr14	10
BCAP31	NM_001256447	chrX	8	LMNB1	NM_001198557	chr5	11
BCS1L	NM_001257344	chr2	8	MCCC1	NR_120640	chr3	19
C19orf12	NM_001256047	chr19	3	MGP	NM_000900	chr12	4
CLCN2	NM_001171088	chr3	23	MLC1	NM_139202	chr22	12
COASY	NM_001042529	chr17	10	MLYCD	NM_012213	chr16	5
COX15	NM_004376	chr10	9	MPV17	NM_002437	chr2	8
COX6B1	NM_001863	chr19	4	NDUFA1	NM_004541	chrX	3
CP	NR_046371	chr3	18	NDUFA10	NM_004544	chr2	10
CSF1R	NM_005211	chr5	22	NDUFA11	NM_001193375	chr19	4
CTC1	NR_046431	chr17	22	NDUFA12	NM_018838	chr12	4
CYP27A1	NM_000784	chr2	9	NDUFA2	NM_001185012	chr5	3
DARS2	NM_018122	chr1	17	NDUFA9	NM_005002	chr12	11
DCAF17	NM_001164821	chr2	12	NDUFAF1	NR_045620	chr15	6
DDC	NM_001242890	chr7	10	NDUFAF2	NM_174889	chr5	4
DLD	NM_001289752	chr7	13	NDUFAF3	NM_199074	chr3	5
EIF2B1	NM_001414	chr12	9	NDUFAF4	NM_014165	chr6	3
EIF2B2	NM_014239	chr14	8	NDUFB3	NM_001257102	chr2	4
EIF2B3	NM_001166588	chr1	10	NDUFS1	NM_005006	chr2	19
EIF2B4	NM_015636	chr2	13	NDUFS2	NM_001166159	chr1	13
EIF2B5	NM_003907	chr3	16	NDUFS3	NM_004551	chr11	7
ERCC6	NM_000124	chr10	21	NDUFS4	NM_002495	chr5	5
ERCC8	NM_000082	chr5	12	NDUFS6	NM_004553	chr5	4
ETHE1	NM_014297	chr19	7	NDUFS7	NM_024407	chr19	8
FA2H	NM_024306	chr16	7	NDUFS8	NM_002496	chr11	7
FAM126A	NM_032581	chr7	11	NDUFV1	NM_007103	chr11	10
FASTKD2	NM_001136193	chr2	12	NDUFV2	NM_021074	chr18	8
FKTN	NM_001079802	chr9	11	NOTCH3	NM_000435	chr19	33
FOLR1	NM_016729	chr11	4	NUBPL	NM_025152	chr14	11
FOXRED1	NM_017547	chr11	11	PANK2	NM_153640	chr20	7
PC	NM_001040716	chr11	23	SAMHD1	NM_015474	chr20	16
PEX1	NM_001282678	chr7	24	SCP2	NM_001007250	chr1	4
PEX10	NM_153818	chr1	6	SDHA	NM_004168	chr5	15
PEX12	NM_000286	chr17	3	SDHAF1	NM_001042631	chr19	1
PEX13	NM_002618	chr2	4	SLC16A2	NM_006517	chrX	6
PEX16	NM_004813	chr11	11	SLC17A5	NM_012434	chr6	11
PEX26	NM_001199319	chr22	5	SOX10	NM_006941	chr22	4
PEX5	NM_001131025	chr12	16	SUMF1	NM_182760	chr3	9
PEX6	NM_000287	chr6	17	SURF1	NM_003172	chr9	9
PHYH	NM_001037537	chr10	8	TRAPPC9	NM_001160372	chr8	23
PLA2G6	NM_003560	chr22	17	TREM2	NM_001271821	chr6	4
PLP1	NM_001305004	chrX	7	TREX1	NM_007248	chr3	2
POLR3A	NM_007055	chr10	31	TUBB4A	NM_001289131	chr19	4
POLR3B	NM_001160708	chr12	28	TUFM	NM_003321	chr16	10
PSAP	NM_002778	chr10	14	TYMP	NM_001113756	chr22	9
RNASET2	NM_003730	chr6	9	TYROBP	NM_003332	chr19	5
RPIA	NM_144563	chr2	9	WDR45	NM_007075	chrX	12

**Table 2:** Demographic and clinical feathers of patients with pathogenic mutations.

Case	Diagnosis	Sex, age (years)	clinical manifestation	Personal History	Developmental milestones	family history	physical examination	Auxiliary examinations	Brain MRI
1	mitochondrial complex I deficiency	Male, 0.9	Motor retardation	G2P2 full-term normal delivery BW:3.05 kg	bristling up head: 4M sit: incapable call mom: 10M	His elder sister (4Y) has a similar brain MRI changes without obvious neurologic symptoms	HC: normal; Hypertonia; hyper-reflexia; Strephexopodia;	Elevated creatine kinase level(297 U/L; normal, <190 U/L); EEG (10M): normal; VEP, BAEP: normal	MRI (11M): Diffuse and symmetric abnormal signal in central and subcortical white matter, hyperintense in the T2 and FLAIR sequences and were hypointense in T1-weighted sequence.
2	mitochondrial complex I deficiency	Male, 0.4	Mental and motor retardation; seizure;	G1P1 full-term normal delivery BW:2.0038 kg	bristling up head: incapable	normal	HC: normal; Hypertonia; Tiptoe;	Increased actate level (4.7 mmol/L; normal, 0.5–2.2 mmol/L); Elevated creatine kinase level (789.7 U/L; normal, <190 U/L); EMG: moderate peripheral demyelinating sensorimotor neuropathy; EEG: slow background; VEP: normal; BAEP: abnormal	MRI (5M): symmetry abnormal signal in bilateral cerebellar hemisphere, hyperintense in the T2 and FLAIR sequences and were hypointense in T1-weighted sequence.
3	mitochondrial complex I deficiency	Female, 3.1	Mental and motor retardation;	G1P1 full-term normal delivery BW:2.5 kg	bristling up head: 6M; sit: 1Y; walk without help: incapable; call mom: 14M	normal	HC: normal; Hypertonia; Horizontal nystagmus	Actate level: normal; Elevated creatine kinase level (320.0 U/L; normal, <190 U/L); EMG: peripheral demyelinating sensorimotor neuropathy;	MRI (2.8Y): symmetry abnormal signal in Periventricular and basal ganglia, hyperintense in the T2 and FLAIR sequences and were hypointense in T1-weighted sequence.
4	MLD	Male, 2.5	extremities weakness; Motor regression;	G2P2 full-term normal delivery BW:2.9 kg	bristling up head: 3^+^M; sit without help: 7M walk without help: 12M call mom: 12M	normal	HC: normal Amyasthnia (1Y); Hypertonia(2Y); Hyper-reflexia(2Y);	EMG: slow sensory and motor nerve conduction velocities; VEP: P100 latency increased; BAEP: latency prolonged; Brain CT: hypointense in Periventricular;	MRI (2.4Y): abnormal signal in periventricular, “tigroid” symptom in T2-weighted sequence;
5	MLD	Femal, 2.4	extremities weakness; Mental regression;	G1P1 full-term Cesarean delivery BW:3.9 kg	bristling up head: 3M sit without help: 7M walk without help: 15M call mom: 13M	normal	HC: normal; Amyasthnia (1.7Y); Hypertonia (2.2Y);	Elevated creatine kinase level (337.5 U/L; normal, <190 U/L); EMG: slow sensory and motor nerve conduction velocities, demyelination and axonal damage; EEG: high voltage and slow wave;	MRI (2.4Y): abnormal signal in periventricular, posterior limbs of internal capsules, hyperintense in the T2 and FLAIR sequences and were hypointense in T1-weighted sequence.
6	MLD	Male, 2.5	Mental regression;	G2P2; full-term; Cesarean delivery; BW:3.1 kg	bristling up head:3^+^M; sit without help: 6^+^M walk without help:14M; call mom: 12M;	normal	HC: normal; Hypertonia (2.1Y);	EMG: demyelination and axonal damage; EEG: high voltage and slow wave, irregular sharp wave in frontal area;	MRI (2Y): abnormal signal in central and subcortical white matter, hyperintense in the T2 and FLAIR sequences and were hypointense in T1-weighted sequence.
7	MLD	Male, 2.1	Mental regression;	G2P1 full-term normal delivery BW:2.9 kg	bristling up head:3M; sit without help: 7M; walk without help:15M; call mom: 12M;	normal	HC: normal; Amyasthnia (1.9Y);	EMG: slow sensory and motor nerve conduction velocities; VEP: P100 latency increased; BAEP: latency prolonged;	MRI (2Y): abnormal signal in the periventricular and the central white matter, “leopard skin”-like change in T2-weighted sequence
8	VWM	Male, 3.5	Seizure; Mental and motor regression;	G3P1 full-term normal delivery BW:3.2 kg	bristling up head:3M sit without help: 7M walk without help:13M call mom: 12M	normal	HC: normal Hypertonia Ataxia	EEG(3Y): Paroxysmal slow wave in sleep stage;	MRI (3.5Y): diffused abnormal signal in the central deep and subcortical white matter, hyperintense in the T2 sequences, hypointense inT1 and FLAIR sequences; MRS: normal.
9	VWM	Femal, 1.2	Seizure; hypotonia	G1P1 full-term Cesarean delivery BW:3.0 kg	bristling up head:3^+^M sit without help: 7M walk without help: incapable call mom: incapable	normal	HC: normal; Hypertonia;	EEG (1.2Y): slow wave in sleep stage; EMG: normal;	MRI (1Y): diffused abnormal signal in the central deep and subcortical white matter, hyperintense in the T2 sequence and hypointense in T1 and FLAIR sequence; MRS: normal.
10	VWM	Male, 2.4	Seizure; Mental and motor regression;	G1P1 full-term normal delivery BW:3.0 kg	bristling up head:3M sit without help: 6M walk without help:12M call mom: 12M	normal	HC: normal; Hypertonia; Amyasthnia;	EEG(2.2Y): Paroxysmal slow wave in REM state; EMG: normal;	MRI (2.4Y): abnormal signal in the white matter of frontal lobe, temporal lobe and periventricular, hyperintense in the T2 sequence and hypointense in T1 and FLAIR sequence; MRS show high Cho crest.
11	MLC	Male, 6.0	macrocephalus; seizure; motor retardation	G1P1 full-term normal delivery BW:2.75 kg	bristling up head:3M sit without help: 6M walk without help:14M call mom: 12M	normal	HC: 57 cm(6Y); Hypertonia; Hyperreflexia; Ataxia;	EEG(6Y): spike waves, sharp waves in REM state, especially in the right temporal lobe;	MRI (6Y): abnormal signal in the white matter of bilateral cerebral hemisphere, hyperintense in the T2 and FLAIR sequence, hypointense in T1 sequence; a 19*13 mm hypointense of right temporal lobe in FLAIR sequence
12	MLC	Femal, 0.7	macrocephalus; Mental and motor retardation;	G1P1 full-term Cesarean delivery BW:2.8 kg	bristling up head: incapable sit without help: incapable	normal	HC: 48 cm(8M); Hypotonia; setting sun eye	EEG(5M): spike waves in REM state, especially in the left temporal lobe;	MRI(6M): cerebral hemispheric swelling, diffuse abnormal signal in the white matter of bilateral cerebral hemisphere, hyperintense in the T2 and FLAIR sequence, hypointense in T1 sequence; a 10*12 mm hypointense of left temporal lobe in FLAIR sequence
13	MLC	Male, 1.7	macrocephalus; seizure; Mental and motor retardation;	G4P2 full-term Cesarean delivery BW:2.57 kg	bristling up head:6M sit without help: 12M walk without help: incapable call mom: 12M	normal	HC: 45.5 cm(4M); Hypotonia;	EEG(1.5M): sharp waves in REM state; EMG: normal;	MRI (1.5Y): abnormal signal in the white matter of bilateral cerebral hemisphere, hyperintense in the T2 and FLAIR sequence, hypointense in T1 sequence; a 6*10 mm hypointense of frontal lobe in FLAIR sequence
14	GLD	Male, 2.8	Mental and motor regression;	G2P2 full-term Cesarean delivery BW:3.0 kg	bristling up head:3 + M sit without help: 6M walk without help: 18M call mom: 13M	normal	HC: normal Hyperreflexia Ataxia;	EEG(2.5Y): sharp waves in left frontal, temporal lobe, slow background waves; EMG: normal;	MRI (2.5Y): symmetry cerebral atrophy, abnormal signal in white matter of brainstem, posterior limb of internal capsule and cerebellum
15	PMD	Male, 4.9	Mental and motor regression;	G2P1 full-term normal delivery BW:3.0 kg	bristling up head:5M sit without help: 14M walk without help: incapable call mom: 13M	normal	HC: normal Hyperreflexia Ataxia;	VEP: P100 latency increased; BAEP: latency prolonged;	MRI (4.5Y): diffuse abnormal signal of white matter, hyperintense in the T2 sequence; MRS: normal.
16	PMD	Male, 2.0	Motor retardation;	G1P1 full-term normal delivery BW:3.0 kg	bristling up head:6M sit without help: 12M walk without help: incapable call mom: 12M	normal	HC: normal; Hypotonia; Nystagmus;	VEP: normal; BAEP: latency prolonged;	MRI (2Y): diffuse abnormal signal of white matter, hyperintense in the T2 sequence; MRS: normal.
17	X-ALD	Male, 7	Motor regression	G2P2 full-term normal delivery BW:3.1 kg	bristling up head:4M sit without help: 8M walk without help: 15M call mom: 12M	normal	HC: normal; Dark complexion; Hypotonia Knee hyperreflexia	ACTH > 440.4 pmol/L(normal:1.6-13.9 pmol/L); Cortisol: normal; PRL: 35.59 mg/ml(normal 3.46-19.0 mg/ml); EEG(7Y): slow background wave; VEP: normal; BAEP: normal;	MRI (7Y): diffuse abnormal signal in callusom and brainstem, hyperintense in the T2 sequence, the signal were intensified in enhanced sequence, “butterfly”-like signal.
18	X-ALD	Male, 7	Progressive vision loss; Motor regression;	G4P2 full-term normal delivery BW:3.0 kg	bristling up head:3M sit without help: 7M walk without help: 12M call mom: 12M	The mother’s brother dead at 10 years old for unclear reason	HC: normal; Dark complexion; Hypotonia Hyperreflexia Ataxia	ACTH, Cortisol, PRL: normal; VEP: normal; BAEP: normal;	MRI (6.5Y): diffuse abnormal signal in callusom and brainstem, hyperintense in the T2 sequence,
19	Zellweger syndrome	Female, 5.8	developmental retardation	G1P1 full-term normal delivery BW:2.7 kg	bristling up head:5M sit without help: 12M walk without help: 2Y call mom: 2Y	normal	HC: normal Hypertonia Hyperreflexia Decreased visual;	EEG: slow background activity with spike-and-wave discharge, localized in the right frontal and temporal region; EMG: normal; VEP: normal; BAEP: latency prolonged;	MRI (5.8Y): abnormal hyperintense in the splenium of corpus callosum, adjacent parieto-occipital white matter, posterior limbs of internal capsules extending to centrum ovale, thalami and upper cervical spinal cord on FLAIR and T2 sequences; Gadolinium enhancement is visible on T1-weighted sequences in internal capsules and anterior commissure.
20	Alexander disease	Male, 0.8	Seizure; developmental retardation	G2P2 full-term Cesarean delivery BW:3.75 kg	bristling up head:5M sit without help: incapable	normal	HC: 46.5(9M); Hypotonia	EEG(9M): sharp wave, slow wave in frontward head; EMG: normal; VEP: normal; BAEP: normal.	MRI (9M): abnormal signal of white matter in frontal and parietal lobe and periventricular, hyperintense in the T2 and FLAIR sequences, hypointense in T1 sequence.

Y = years; M = months; BW = birth weight; HC = head circumference; GDD = global developmental delay; EEG = electroencephalograms; EMG = electromyography; VEP = visual evoked potential; BAEP = brain auditory evoked potentials; MRI = magnetic resonance imaging; FLAIR Sequence = fluid-attenuated inversion recovery sequences; All the acronym of Diagnosis can see in the article.

**Table 3:** Gene identified by targeted capture and MPS in atypical leukoencephalopathy patients.

Probands	Sex, age (years)	Genomic coordinates^a^	Reference reads	Variant reads	Mutation gene	cDNA	Protein	HGMD reported or not	de novo/inherited	ExAC_MAF	1000 genomes	SIFT	Mutation Taster	PolyPhen-2 HumVarscore
1	M, 0.9	chr11:67376961 C > T	130	128	*NDUFV1*	c.338C > T (NM_001166102.1)	p.Pro113Leu	Unreported	Paternal	0.00004118	—	Deleterious low confidence(0)	Disease causing	Probably damaging (1)
		chr11:67377072 G > A	139	123	*NDUFV1*	c.449G > A (NM_001166102.1)	p.Arg150Gln	Unreported	Maternal	—	—	Deleterious low confidence(0)	Disease causing	Probably damaging (0.989)
2	M, 0.4	chr15:41688980 T > C	178	190	*NDUFAF1*	c.278A > G (NM_016013.3)	p.His93Arg	Reported	Paternal	—	—	Deleterious (0.01)	Disease causing	Benign(0.043)
		chr15:41689011 C > T	125	115	*NDUFAF1*	c.247G > A (NM_016013.3)	p.Asp83Asn	Reported	Maternal	0.0000329	—	Deleterious (0.03)	Polymorphism	Benign(0.349)
3	F, 3.1	chr1:161172233 C > A	27	27	*NDUFS2*	c.58C > A (NM_001166159.1)	p.Pro20Thr	Unreported	Maternal	0.087	0.0865	Tolerated low confidence(0.34)	Polymorphism automatic	Benign(0.001)
		chr1:161180394 C > T	86	64	*NDUFS2*	c.880C > T (NM_001166159.1)	p.Arg294Trp	Unreported	Paternal	—	—	Deleterious(0)	Disease causing	Probably damaging (1)
4	M, 2.5	chr22:51065317 A > G	60	64	*ARSA*	c.371T > C (NM_001085428.2)	p.Leu124Pro	Unreported	Paternal	—	—	Deleterious(0)	Disease causing	Probably damaging (0.991)
		chr22:51065757 C > A	52	33	*ARSA*	c.44G > T (NM_001085428.2)	p.Gly15Val	Unreported	Maternal	—	—	Tolerated(0.2)	Polymorphism	Benign(0.116)
5	F, 3	chr22:51063758 -51063759insC	347	315	*ARSA*	c.1087_1088insC (NM_001085428.2)	p.Gly363 Alafs*124	Unreported	Maternal	—	—	—	Disease causing	—
		chr22:51066021 -51066022insCA	196	188	*ARSA*	c.187_188insCA (NM_000487.5)	p.Asp63 Alafs*18	Unreported	Paternal	—	—	—	Disease causing	—
6	M, 2.5	chr22:51065689 C > T	0	47	*ARSA*	c.370G > A (NM_000487.5)	p.Gly124Ser	Reported	Paternal/Maternal	0.00001668	—	Deleterious (0.01)	Disease causing automatic	Possibly damaging(0.895)
7	M, 2.1	chr22:51063674-51063674insC	20	28	*ARSA*	c.1170dupC (NM_001085428.2)	p.Ser391 Glnfs*96	Unreported	Paternal	—	—	—	Disease causing	—
		chr22:51065133 C > T	38	21	*ARSA*	c.740G > A (NM_000487.5)	p.Gly247Glu	Unreported	Maternal	—	—	Deleterious(0)	Disease causing	Probably damaging(0.999)
8	M, 3.5	chr2:27587620 C > T	0	142	*EIF2B4*	c.1334G > A (NM_015636.3)	p.Arg445His	Reported	Paternal/Maternal	—	—	Deleterious(0)	Disease causing	Probably damaging(0.996)
9	F, 1.2	chr3:183857908 G > A	0	1136	*EIF2B5*	c.806G > A (NM_003907.2)	p.Arg269Gln	Reported	Paternal/Maternal	—	—	Deleterious (0.04)	Disease causing	Benign(0.402)
10	M, 2.4	chr3:183858366 G > C	417	549	*EIF2B5*	c.1004G > C (NM_003907.2)	p.Cys335Ser	Reported	Paternal	—	—	Tolerated (0.23)	Disease causing	Benign(0.084)
		chr3:183860329 A > G	1001	796	*EIF2B5*	c.1484A > G (NM_003907.2)	p.Tyr495Cys	Reported	Maternal	0.000008326	—	Deleterious(0)	Disease causing automatic automatic	Possibly damaging(0.621)
11	M, 6	chr22:50502592-50502599del	3	3	*MLC1*	c.924_929del (NM_139202.2)	p.Leu309_Leu310del	Reported	Paternal	—	—	—	polymorphism	—
		chr22:50521562 G > A	70	94	*MLC1*	c.218G > A (NM_015166.3)	p.Gly73Glu	Reported	Maternal	—	—	Deleterious low confidence (0)	Disease causing	Probably damaging(1)
12	F, 0.7	chr22:50521562 C > T	0	88	*MLC1*	c.218G > A (NM_015166.3)	p.Gly73Glu	Reported	Paternal	—	—	Deleterious low confidence (0)	Disease causing	Probably damaging(1)
13	M, 1.7	chr22:50521562 C > T	0	388	*MLC1*	c.218G > A(NM_015166.3)	p.Gly73Glu	Reported	Paternal/Maternal	—	—	Deleterious low confidence (0)	Disease causing	Probably damaging(1)
14	M, 2.8	chr14:88411981 G > A	53	33	*GALC*	c.1586C > T (NM_000153.3)	p.Thr529Met	Reported	*De novo*^b^	0.000066225	—	Deleterious (0.01)	Disease causing	Probably damaging (0.995)
		chr14:88417067 G > A	29	33	*GALC*	c.1187G > A (NM_000153)	p.R396Q	Unreported	Paternal	—	—	Deleterious(0)	Disease causing	Probably damaging (0.962)
15	M, 4.9	Duplication	/	/	*PLP1*	/	/	Reported	*De novo*	—	—	—		—
16	M, 2	chrX:103043377 T > C	0	348	*PLP1*	c.634T > C (NM_000533.4)	p.Trp212Arg	Reported	Maternal	—	—	Deleterious(0)	Disease causing	Probably damaging (0.999)
17	M, 7	chrX:153002662 T > A	0	33	*ABCD1*	c.1445T > A (NM_000033.3)	p.Val482Asp	Unreported	*De novo*	—	—	Deleterious(0)	Disease causing	Benign(0.013)
18	M, 7	chrX:152991011 A > C	5	181	*ABCD1*	c.290A > C (NM_000033.3)	p.His97Pro	Reported	Maternal	—	—	Deleterious(0)	Disease causing	Probably damaging (0.99)
19	F, 5.8	chr6:42932599 G > A	59	62	*PEX6*	c.2735C > T (NM_000287.3)	p.Ala912Val	Unreported	Paternal	0.000008326	—	Deleterious(0)	Disease causing	Probably damaging (1)
		chr6:42937459: 42insT	38	26	*PEX6*	c.1313dupT (NM_000287.3)	p.Glu439 Glyfs*6	Unreported	Maternal	0.000008327	—	—	Disease causing	—
20	M, 0.8	chr17:42992605 T > A	63	33	*GFAP*	c.250A > T (NM_001131019.2)	p.Ile84Phe	Unreported	*De novo*	—	—	Deleterious(0)	Disease causing	Probably damaging (0.97)

F = Female; M = Male; cDNA = complementary DNA; HGMD = The human gene mutation database.

^a^hg19.

^b^It’s not sure whether the de *novo* mutation in patient 14 was in maternal allele or not.
